# Circulating Tumor DNA in Cervical Cancer: Clinical Utility and Medico-Legal Perspectives

**DOI:** 10.32604/or.2025.072176

**Published:** 2025-12-30

**Authors:** Abdulrahman K. Sinno, Aisha Mustapha, Navya Nair, Simona Zaami, Lina De Paola, Valentina Billone, Eleonora Conti, Giuseppe Gullo, Pasquale Patrizio

**Affiliations:** 1 Division of Gynecologic Oncology, Sylvester Comprehensive Cancer Center, University of Miami Miller School of Medicine, Miami, FL 33136, USA; 2 Department of Anatomical, Histological, Forensic and Orthopedic Sciences, Sapienza University of Rome, Rome, 00161, Italy; 3 Department of Obstetrics and Gynecology, AOOR Villa Sofia–Cervello, University of Palermo, Palermo, 90127, Italy; 4 Division of Reproductive Endocrinology & Infertility, University of Miami Miller School of Medicine, Miami, FL 33136, USA

**Keywords:** Circulating tumor DNA, circulating tumor human papillomavirus, cervical cancer, medico-legal implications

## Abstract

Cervical cancer related to human papillomavirus (HPV) is a leading cause of cancer-related mortality among women worldwide. Cancer cells release fragments of their DNA, known as circulating tumor DNA (ctDNA), which can be detected in bodily fluids. A PubMed search using the terms “ctHPV” or “circulating tumor DNA” and “cervical cancer”, limited to the past ten years, identified 104 articles, complemented by hand-searching for literature addressing medico-legal implications. Studies were evaluated for relevance and methodological quality. Detection and characterization of circulating tumor HPV DNA (ctHPV DNA) have emerged as promising tools for assessing prognosis and disease recurrence in cervical cancer. Detection techniques include polymerase chain reaction (PCR), digital droplet PCR (ddPCR), and next-generation sequencing (NGS). This review summarizes current knowledge on ctHPV DNA in cervical cancer and explores its clinical and medico-legal implications, including management of discordant results, diagnostic errors, liability, and data protection compliance.

## 1 Introduction

Worldwide, cervical cancer is the fourth most common and most lethal cancer in women [1]. In 2020, there were 604,127 new cervical cancer cases and 341,831 cervical cancer deaths in the world [1]. About 90% of cases of cervical cancer occur in lower-and middle-income countries (LMICs) [1], with most cases presenting in late stages. High-income countries, on the other hand, have seen a 70% reduction in the incidence and mortality due to organized screening programs and HPV vaccination [1,2].

Early-stage disease is usually treated surgically, with sentinel lymph node (SLN) mapping improving staging accuracy and reducing morbidity compared to lymphadenectomy [3]. Locally advanced cervical cancer is treated with chemotherapy/immunotherapy and external beam radiation followed by high-dose brachytherapy, while metastatic or recurrent disease relies on systemic chemotherapy, with or without checkpoint inhibitors [4]. Despite advancements in cervical cancer treatment, recurrence rates remain high [5]. The detection of recurrence relies on patient symptoms and pelvic examination, followed by imaging when either is positive. Ultimately, a tissue biopsy is needed to confirm the diagnosis of recurrence. There is currently no universally accepted serologic biomarker for detection, monitoring of treatment response, or determination of recurrence. Moreover, in LMICs, where the burden of cervical cancer is the highest, access to imaging and tissue diagnosis remains challenging.

The overwhelming majority of cervical cancer is caused by the human papillomavirus (HPV) [6]. HPV, a non-enveloped and double-stranded DNA virus, belongs to the family of Papillomaviridae. Eight high-risk (oncogenic) HPV types 16, 18, 31, 33, 35, 45, 52, 58 account for approximately 90% of invasive cervical carcinomas positive for HPV DNA [1,7]. Within the virus structure, the genetic material is enclosed by an icosahedral capsid composed of L1 and L2, which are major and minor structural proteins, respectively.

The viruses are highly tissue-specific and infect both mucosal and cutaneous epithelium [8]. The two major oncogenes, E6 and E7, inhibit the functions of tumor suppressor genes p53 and pRb in normal cervical epithelial cells and cause abnormal proliferation, leading to the development of genital warts and cancer [2]. Most HPV infections are cleared by the immune system within 3 years; however, persistent HPV infection and expression of oncoproteins E6 and E7 lead to viral incorporation into the host DNA, progression to dysplastic cells, and ultimately invasive carcinoma [9,10].

Different biomarkers for cervical cancers have been studied in the past. The squamous cell carcinoma antigen (SCCA) is a tumor-specific antigen, first isolated from cervical tissue in the 1970s [11], and extensively studied as a biomarker, but its role in diagnosis and prognosis remains to be established [12]. The efficacy of combined Positron Emission Tomography/Computed Tomography (PET/CT) with serum SCCA in the diagnosis of early recurrent cervical cancer is higher than that of either PET/CT or serum SCCA alone [13,14]; however, there is still insufficient evidence about the clinical utility of this marker.

In this article, we will review the existing literature on circulating cell-free DNA and specifically the role of circulating tumor HPV DNA in cervical cancer treatment, and discuss its potential implications in medico-legal and ethical contexts.

## 2 Material and Methods

### 2.1 Methods

**Figure 1:**
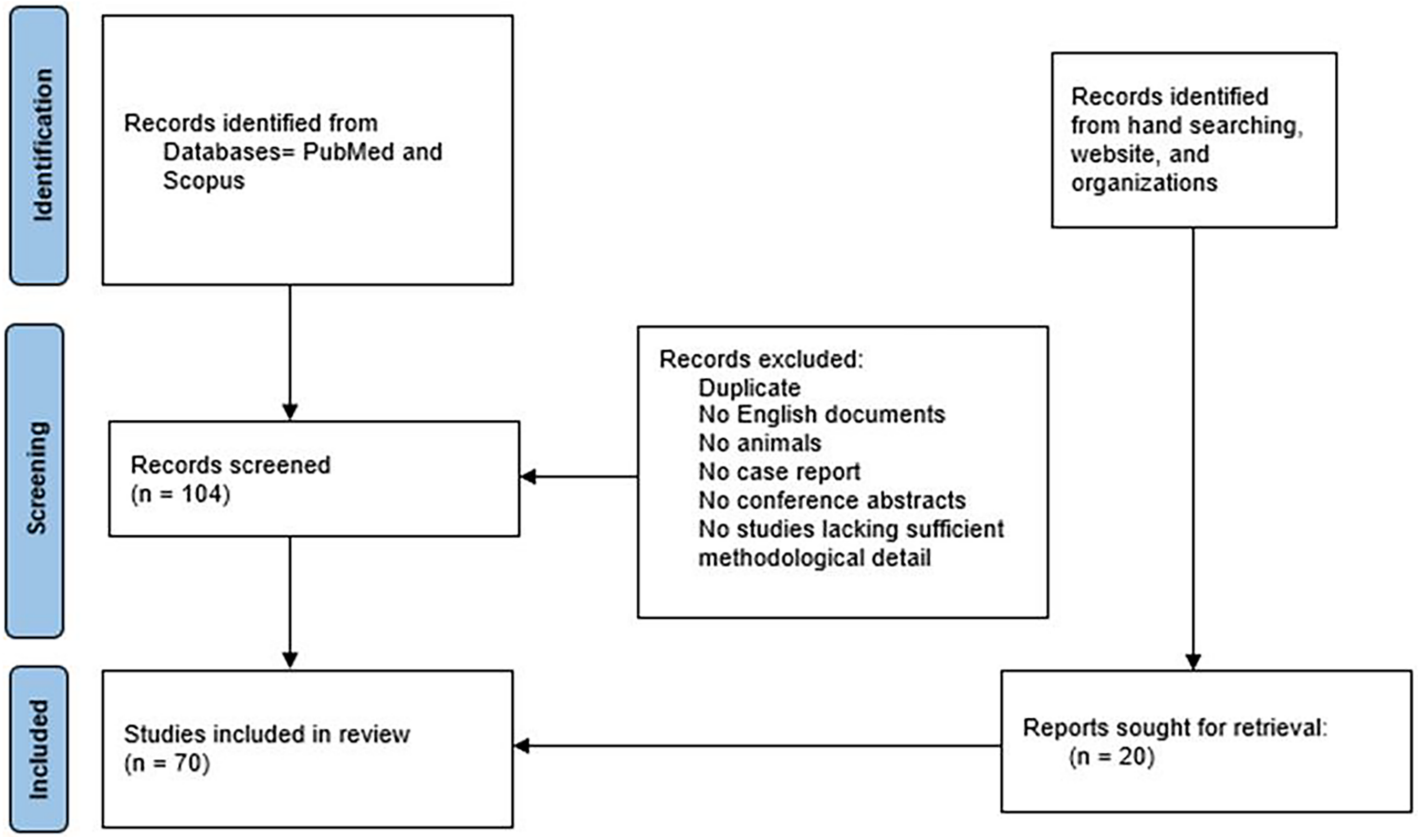
PRISMA flow chart

This is a narrative review with a structured literature search, and it was conducted following principles adapted from the PRISMA guidelines to ensure transparency and reproducibility. We performed an electronic database search using PubMed and Scopus, limited to the past ten years, with the search terms: “*ctHPV*” or “*circulating tumor DNA*” and “*cervical cancer*”. This search yielded 104 articles. Additionally, we manually screened the reference lists of these articles and conducted hand-searching to identify literature with medico-legal, regulatory, or ethical relevance that may not have been captured in the initial query. We included original research articles and reviews published in peer-reviewed journals, in English, with full-text availability. We excluded conference abstracts, non-English publications, animal or cell line studies without ctHPV DNA data from human cervical cancer patients, and studies lacking sufficient methodological detail. The assessment was conducted by two reviewers independently, with any disagreements resolved through discussion or by involving a third reviewer. For the medico-legal and ethical aspects, we specifically searched for data related to informed consent procedures, data privacy policies, case reports, and litigation references. Titles and abstracts were screened independently by two authors, and full-text articles meeting the inclusion criteria were subsequently assessed. Following this process, 70 articles were included in the final review, as shown in Fig. 1.

The aim of this review is to summarize the current knowledge and recent advancements in the study of circulating tumor HPV DNA (ctHPV DNA) in cervical cancer, and to explore its potential clinical applications as well as possible medico-legal implications.

### 2.2 Methodological Limitations of the Included Studies

The studies included present some methodological limitations that may affect the interpretation of the results. Among these, many studies had small sample sizes, reducing the generalizability of the data; some were retrospective in design, exposing the results to potential selection and information biases; in several cases, a control group was lacking, making it difficult to distinguish the specific effects of the intervention or exposure from the natural development of the phenomena studied. Moreover, differences in inclusion criteria, measurement instruments, and follow-up periods contributed to methodological heterogeneity, which inevitably was reflected in our study, presenting the same limitations (see Section 4.4).

## 3 Results

### 3.1 Liquid Biopsy and Circulating Cell-Free DNA

Traditionally, confirmation of tumor presence or recurrence is with pathologic tissue assessment from surgical resection specimens or guided biopsies. Drawbacks of this diagnostic modality are related to (1) the invasiveness of the procedure, (2) the risk of complications, (3) the lack of ability to repeat this indefinitely, (4) the need for the tumor to be large enough to be successfully biopsied, and (5) the need for the tumor location to be accessible to biopsy. Liquid biopsy is a noninvasive approach for assessing tumors without the need for tissue sampling. It offers the benefit of ease of repeat testing as molecular tumor profiles change both over time and in response to treatments [15]. It also allows for the assessment of various types of circulating genetic entities, such as circulating tumor cells, cell-free DNA (cfDNA), exosomes (small vesicles carrying RNA and DNA), and circulating microRNA [15]. It allows for assessment of heterogeneous tumor DNA [16]. Isolation of circulating cfDNA has been well replicated and studied [17–19] in other HPV associated tumors such as head and neck, and anal cancers [20,21].

Cell-free DNA (cfDNA) is a short segment of DNA that is released into bodily fluids, including blood, plasma, saliva, cerebrospinal fluid, and amniotic fluid [22] either by active secretion or as a result of cellular apoptosis or necrosis. The size of cfDNA segments depends on the mechanism triggering their release, and they can range from 180 base pairs during cellular apoptosis to 10,000 base pairs during necrosis [23]. Typically, macrophage immune activity degrades these fragments of DNA so that healthy individuals have low concentrations of cfDNA, less than 10 ng/mL [24]. In patients with cancer, however, the levels of circulating cfDNA are typically greater than 1000 ng/mL secondary to the rapid turnover of tumor cells.

As such, cfDNA, which is derived from tumor cells, is referred to as ctDNA (circulating tumor DNA) [24]. ctDNA is typically shorter than physiologic cfDNA, and this aids in the detection and differentiation of ctDNA. Furthermore, ctDNA has a half-life of less than 20 min, making it a real-time reflection of current disease status [25,26]. The majority of cfDNA originates in blood cells (85%), 10% from vascular endothelial cells, 1% from the liver cells, with the remaining derived from other cells, including cancer cells [27].

The presence of integrated HPV DNA in host cells has been demonstrated with progressively increasing frequency from normal cervical epithelium to cervical intraepithelial neoplasia (CIN) to invasive cervical cancer [28]. The unique non-human viral sequence, the numerous copies circulating after amplification, and the presence of either integrated HPV genomes and/or multiple episomal copies in cancer cells make ctHPV DNA a favorable marker for detecting and monitoring cervical cancer [29]. To ensure a precise and clinically relevant qualitative and quantitative measure of ctHPV DNA in blood samples, an assay with sufficient specificity and sensitivity is essential. Mutation detection is the liquid biopsy target used in non-HPV-related cancers and offers benefits and drawbacks over ctHPV DNA detection, which will be discussed in this manuscript.

### 3.2 Circulating Tumor HPV DNA Testing Assays

Circulating tumor HPV DNA (ctHPV DNA) is an attractive biomarker for cervical cancer because it is implicated in almost all cases of the disease and exists in multiple copies per cell [30]. Furthermore, because the HPV integrates into the host genome to cause tumorigenesis, integrated HPV sequences can be easily differentiated from normal non-infected host tissue cell-free DNA, making them excellent targets for liquid biopsy in HPV mediated cancers [31]. Most studies evaluating cfDNA in cervical cancer involve the detection of ctHPV DNA, with many studies focusing on HPV16/18, which is present in 70% of cervical cancers [32]. By using primers and probes that specifically bind to E6, E7 and L1 sequences, it is possible to design specific and sensitive targeted quantitative polymerase chain reaction (PCR) assays [33]. Detection methods include conventional PCR [31,34–36], quantitative PCR (qPCR) [37–40], and digital droplet PCR (ddPCR) [29,41–44] and next-generation sequencing (NGS) [45,46].

Earlier studies described poor sensitivity in detecting ctHPV DNA, ranging from 6.9% to 30% [31,34–36,38,39,47]. Possible reasons for this low sensitivity include the poor sensitivity of the PCR method used for these studies to detect the minuscule fragments of ctHPV DNA. Real-time qPCR allowed for higher detection rates than conventional PCR, but this method continued to be limited when low copies of the target gene are present [48,49]. The ddPCR platform can perform absolute quantification of ctHPV DNA copy numbers in blood, serum, or plasma, using massive parallel sequencing with error suppression and ultra-sensitive targeted methods [33]. This allows for detection of HPV DNA at very low limits of detection and with high specificity (97%–100%) and high sensitivity (89%–98%) [50]. Using ddPCR and specifically targeting E7 and L1 genes in cfDNA, investigators have been able to correlate elevated pretreatment viral load with higher risks of recurrence and lower overall survival [42,51]. Jeannot et al. demonstrated an increase in the detection of HPV DNA from 48/70 when using qPCR to 61/70 when using ddPCR in cervical cancer patients. A practical drawback to using ddPCR is the requirement of a priori knowledge of HPV genotype and infection status to design a probe specific to the HPV infection at hand [29].

With increasing cancer stage, there is a directly proportional increase in the level of ctHPV DNA [52]. In cancer patients, levels of ctHPV DNA increase to an average of 29 ng/mL plasma in stages I-III [53], and up to 1000 ng/mL in metastatic cancer [54]. It has been shown in non-small cell lung cancer that the amount of ctHPV DNA is often affected not just by the tumor size but also by the metabolic tumor burden [55,56]. The amount of ctHPV DNA levels has been shown to correlate with liver metastasis, and to a lesser extent, with lymph node and peritoneal metastasis [57–61].

### 3.3 Circulating HPV DNA for Premalignant Lesions

Even though ctHPV DNA can be found in cervical cancer, data are still conflicting regarding the presence of this marker in the circulation of individuals with precancer lesions. Numerous studies could not find this marker in the serum of patients with premalignant lesions [35–37,43,52] despite confirmed HPV-positive genital lesions [29,39]. Dong et al. found that 1/57 patients with cervical carcinomas *in situ* had circulating HPV DNA in their blood [34]. Two studies found the presence of circulating HPV DNA in patients with high-grade squamous intraepithelial lesion (HSIL) and low-grade squamous intraepithelial lesion (LSIL) of the cervix, at 6/18, 33% and 9/18, 50% for HSIL and 3/15, 20% and 16/52, 31% for LSIL [62], with poor correlation between HPV DNA-status in plasma and in the cervix. The majority of such studies that detected circulating HPV DNA in precancers were conducted before 2016, with some methodological limitations. However, even the more recent studies using more sensitive methods like ddPCR have also not found ctHPV DNA in the circulation of any patients with premalignant lesions [33,43,52].

### 3.4 ctHPV DNA in Women without Cervical Pathology

It is the persistence of HPV infection and the immune system’s inability to clear the infection that results in the malignant transformation of cervical cells. As such, the question arises whether transient HPV infections can result in the presence of cfHPV DNA even without the presence of a cervical lesion. A study by Yang et al., which included 50 cervical cancer patients and 96 patients with no cervical lesion [40]. cfHPV16/18-DNA was detected in 56% of cancer patients and in 14.6% patients with no cervical lesions [40]. Many other studies, however, found that the rate of cfHPV DNA in healthy patients was less than 2% [43,63].

### 3.5 Mutation Analysis of ctHPV DNA in Cervical Cancer

Integration of HPV DNA into cervical cells is known to cause chromosomal abnormalities, DNA damage, and chromosomal alterations. These alterations are highly variable among tumors, but most frequently, the ERBB2/PI3K/AKT/mTOR pathway is affected [64–67]. Chung et al. assessed the feasibility of detecting two PIK3CA mutations, p.E542K and p.E545K, in cell-free DNA (cfDNA) extracted from pretreatment plasma of 177 patients using ddPCR [67]. PIK3CA mutations were only detected in cfDNA in 22.2% of patients. Mutation detection status was significantly associated with tumor size (*p* < 0.01) and significantly associated with decreased disease-free survival and overall survival (*p* < 0.05).

While PCR is efficient in detecting known mutations, next-generation sequencing allows for a larger panel of mutation detection and potentially allows for the identification of new mutations or tumor-specific mutations. In a study by Lee et al., utilizing NGS of 24 different mutations, 75 percent of 24 cervical cancer patients were found to harbor these mutations, and in greater than 75% of the time these mutations were found in ZFHX3, KMT2C, and KMT2D [68]. In a study of 10,000 Chinese cancer patients, including 123 cervical cancer patients, Zhang et al. utilized deep sequencing of greater than 1000 genes, noted that this method resulted in a sensitivity of greater than 70 percent in metastatic cervical cancer patients and a 60.9% sensitivity in stage I-III patients [69]. PIK3CA mutations were found most in cervical cancer patients, and more than 25% of patients had a high tumor mutational burden.

### 3.6 Prognostic and Predictive Role of ctHPV DNA in Cervical Cancer

ctHPV DNA has emerged as a biomarker with prognostic and predictive implications in cervical cancer. From a prognostic perspective, ctHPV DNA detection has been correlated with tumor stage, tumor burden, and survival outcomes [30,70]. Persistently elevated ctHPV DNA levels after definitive therapy have been associated with increased risk of recurrence and inferior progression-free survival, whereas rapid ctHPV DNA clearance during treatment correlates with a favorable prognosis [71]. Importantly, longitudinal ctHPV DNA monitoring enables the identification of minimal residual disease (MRD) and can anticipate radiological or clinical relapse by several months, providing a window of opportunity for early therapeutic intervention.

From a predictive standpoint, ctHPV DNA dynamics during therapy may serve as an early marker of treatment efficacy. Reductions in ctHPV DNA levels during the initial phases of chemoradiation have been shown to predict treatment response, while stable or rising ctHPV DNA levels suggest radioresistance or chemoresistance. This real-time monitoring capability offers potential advantages over conventional imaging, which may underestimate or delay recognition of treatment failure [29]. Furthermore, genomic profiling of ctHPV DNA may identify emerging resistance mechanisms (e.g., alterations in DNA repair or immune evasion pathways), providing actionable insights for the use of targeted therapies or immunotherapeutic strategies in recurrent and metastatic disease [3]. Collectively, ctHPV DNA analysis in cervical cancer represents not only a minimally invasive diagnostic approach but also a tool with significant prognostic and predictive value, capable of refining risk stratification, enabling early detection of recurrence, and supporting precision oncology approaches in clinical practice.

## 4 Discussion

### 4.1 Use of Cell-Free DNA in Monitoring of Cervical Cancer Treatment

An increasing number of studies confirm that persistent ctHPV DNA after treatment confers a poor prognosis and that lower levels are associated with improved clinical outcomes [52]. In a multicenter validation study, Han et al. prospectively followed 70 patients with cervical cancer after definitive chemoradiation with plasma ctHPV DNA levels quantified using both ddPCR and HPV-seq [72]. Patients were assessed at baseline, end of treatment, 4–6 weeks post-treatment, and 3 months post-treatment. The primary objective was to detect a difference in 2-year progression-free survival (PFS) between those with detectable vs. undetectable ctHPV DNA after treatment. ctHPV DNA was detected in 70/75 patients. In 3 of the 5 patients where ctHPV DNA was not detected in the blood, the pathologic specimens were found to be HPV negative. Interestingly, the median time from the first subsequent positive test to clinical detection of relapse was 5.8 months. Patients who had persistently detectable ctHPV DNA after treatment had a worse 2-year PFS as compared to patients with undetectable levels.

In a study by Mittelstadt et al., next-generation sequencing targeting 13 different types of HR HPV DNA was used to detect ctHPV DNA [44]. A significant correlation between tumor burden and ctHPV DNA levels was found in 17/17 patients with advanced-stage disease. Of the patients with early-stage disease, 5/9 had ctHPV DNA detected (Stage 1A-1B2). The subsequent levels of ctHPV DNA also correlated with treatment response, and in one patient, an elevated level correlated with relapse. Another study of 94 patients with HPV 16 or HPV 18 positive cervical cancers used ddPCR to successfully identify circulating HPV 16/18 DNA in 59/94 patients prior to treatment. Positive detection correlated with higher stage and HPV burden within the primary tumor. They also found that undetectable post-treatment ctHPV DNA was associated with longer progression-free survival. In this study, the median time from persistent ctHPV DNA detection to recurrence was 10 months [30]. Cabel et al. analyzed blood and tumor samples from 55 patients with HPV-positive locally advanced cervical cancer treated with chemotherapy and radiation and successfully detected ctHPV DNA in 38/55 patients by ddPCR. Detection of ctHPV DNA post-treatment was associated with worse progression-free and overall survival, and levels of ctHPV DNA also correlated with advanced stage and high intra-tumoral HPV burden [73]. In a Swedish study, Sivars et al. [52] developed a ddPCR assay for the E7 gene of HPV 16, 18, and 45. Those specific HPV strains were selected as they account for more than 85% of cervical cancer HPV genotypes in Sweden. The sensitivity of detection of ctHPV DNA was 94.4% for patients with locally advanced cervical cancer. Larger tumors had significantly higher concentrations of ctHPV DNA than smaller tumors. While pretreatment ctHPV DNA levels did not correlate with survival, patients with persistent ctHPV DNA at the end of their treatment had statistically worse progression-free and overall survival.

A prospective multicenter study comparing the accuracy of ctHPV DNA monitoring with PET/CT monitoring for patients with cervical cancer who have completed treatment demonstrated that the area under the ROC (AUC) for detection was 77% and 60%, respectively (*p* = 0.008), concluding that a 3-month post-treatment ctHPV DNA was more accurate than 3-month post-treatment PET/CT imaging for detecting residual disease. The authors of this study also confirmed that patients with undetectable ctHPV DNA after treatment had a 92% progression-free survival as opposed to 50% progression-free survival in patients with detectable levels [72]. A subsequent study used an NGS-based test called ‘panHPV detect’ based on the genotypes of the eight high-risk HPV genotypes associated with cervical cancers (16, 18, 31, 33, 35, 45, 52, and 58). The assay was able to detect recurrence in 3 patients who exhibited a complete radiologic response [44].

Interestingly, four patients with persistent disease on imaging but a negative ctHPV DNA assay did not show any signs of relapse [44].

In Table 1, we have summarized selected studies from recent years that have evaluated the use of ctHPV DNA in predicting and detecting cancer recurrences, and which, in our view, provide meaningful evidence supporting this approach.

**Table 1:** Studies evaluating the use of ctHPV DNA in predicting and detecting cancer recurrences

Ref.	Sample size (N)	HPV types	Sample type	Detection method	Follow-up period	Sensitivity	Specificity	Clinical utility
Han et al. 2024 [72]	70	Thirty-eight high-risk HPV types	Plasma	ddPCR, HPV seq	a median follow-up of 2.2 (range, 0.5–5.5) years	*At baseline: 100% for HPV seq vs. 93% for ddPCR* *At the end of therapy: 57 for ddPCR vs. 81 for HPV seq*	*At the end of therapy: 68 for ddPCR vs. 51 for HPV-seq*	*Predictive biomarker:* time from first ctHPV DNA to clinical recurrence was 5.9 months
Mittelstadt et al. [44]	17	16, 18, 31, 33, 35, 39, 45, 51, 52, 56, 58, 59, 68	Plasma	NGS	Range 1–2 years	*At baseline: 85%* *At the end of therapy: NA*	*NA*	*Predictive biomarker*: ctHPV DNA level decrease correlated with clinical response
Jeannot et al. [30]	94	16, 18	Serum	ddPCR	18 months	*At baseline: 63%* *At the end of therapy: 89.7%*	*At the end of therapy: 92.9%*	*Predictive biomarker:* time from first ctHPVDNA to clinical recurrence was 10 months
Cabel et al. [73]	55	16, 18, 31, 33, 35, 45, 52, 58, 73	Serum/plasma	ddPCR	a median follow-up of 49.9 months (range: 10–130 months) for the retrospective cohort and 37 months (range: 15–52 months) for the prospective cohort	*At baseline: 69%* *At the end of therapy and/or FU: 60%*	*AT the end of therapy: 89%*	*Prognostic biomarker:* Detectable ctHPV DNA at the end of chemoradiation was associated with lower PFS
Sivars et al. [52]	33	16, 18, 45	Plasma	ddPCR	a mean of 24.8 months (range 16.9–33.6 months) for patients with locally advanced cervical cancer; a mean of 14.4 months (range 8.2–30.1 months) for patients with biopsy-verified early-stage cervical cancer	*NA*	*NA*	*Prognostic biomarker:* Higher levels of pretreatment ct HPV DNA correlated with worse PFS
Han et al. 2018 [74]	19	16, 18, 31, 33, 35, 39, 45, 51, 52, 56, 58, 59, 66, 68	Plasma	qPCR, ddPCR	Median follow-up was 24 months (range, 18 to 30 months)	*At baseline: 100%* *At the end of therapy: 75%*	*At the end of therapy: 80%*	*Prognostic biomarker:* Detectable ctHPV DNA at the end of chemoradiation was associated with lower PFS

Note: ctHPV DNA, circulating tumor HPV DNA; ddPCR, digital droplet polymerase chain reaction; NGS, next-generation sequencing; qPCR, quantitative polymerase chain reaction; PFS, progression-free survival; NA, not applicable.

### 4.2 Clinical Implications

The integration of ctHPV DNA monitoring into post-treatment surveillance protocols for cervical cancer has the potential to enhance clinical decision-making. A practical approach to the integration of ctHPV DNA into post-treatment surveillance can be conceptualized as a stepwise algorithm. At the completion of definitive therapy, a baseline ctHPV DNA assessment should be obtained in parallel with standard clinical evaluation and, when indicated, imaging. An early follow-up measurement, typically performed 4–6 weeks after treatment, can provide the first indication of molecular response [73,75,76]. Patients with undetectable ctHPV DNA at this stage may continue with guideline-based surveillance, reserving imaging for cases with clinical suspicion. In contrast, those with detectable ctHPV DNA should undergo repeat testing at three months, with consideration of early imaging to evaluate for residual or recurrent disease.

At the three-month post-treatment evaluation, persistently undetectable ctHPV DNA is highly reassuring and supports continuation of standard follow-up intervals. Conversely, persistent or rising ctHPV DNA strongly suggests residual disease or early recurrence and should prompt timely imaging, multidisciplinary assessment, and, when feasible, biopsy confirmation. During long-term surveillance, serial ctHPV DNA measurements at intervals of 3–6 months during the first two years can further refine risk stratification. Patients who remain ctHPV DNA–negative may be considered for less intensive imaging strategies, whereas those who convert from negative to positive results should be investigated promptly, even in the absence of radiologic abnormalities, given the consistent lead time of ctHPV DNA detection over clinical recurrence [77].

### 4.3 Medico-Legal and Ethical Frameworks

The introduction of ctHPV DNA quantification as a clinical monitoring tool for patients with cervical cancer, as with other gynecological malignancies, such as breast cancer [78–80], alongside other more or less established methods, represents a significant advancement in patient management but simultaneously raises important medico-legal and ethical issues [75,81]. Numerous studies have shown that persistent ctHPV DNA after treatment is associated with a poor prognosis and can anticipate the clinical diagnosis of relapse by several months compared to traditional imaging methods. This diagnostic anticipation may offer healthcare professionals and patients the opportunity to intervene promptly by modifying therapies or performing additional diagnostic tests, which together potentially improve clinical outcomes; naturally, this also entails substantial responsibilities for healthcare providers, patients, and the healthcare system [82–84], similar to what occurs in many other medical fields, including innovative sectors employing new technologies such as artificial intelligence in medicine [85–87]. From a strictly medico-legal perspective, the routine use of ctHPV DNA in follow-up requires clear definitions of diagnostic and therapeutic protocols, since the detection of ctHPV DNA in the absence of clinical or radiological evidence of residual disease can generate clinical decision-making uncertainties and, naturally, psychological challenges for the patient [88,89].

This situation therefore opens an ethical as well as medico-legal debate: failure to perform further investigations or treatments could be interpreted as medico-legal negligence and thus be punishable, as well as being ethically controversial; conversely, performing invasive and sometimes aggressive investigations and treatments solely based on molecular positivity could constitute overtreatment, potentially leading to iatrogenic harm with subsequent medico-legal disputes and economic repercussions for healthcare institutions and society, especially in public health systems compared to no-fault compensation systems [90,91]. These applications thus require a careful balance between the precautionary principle and protecting patients from unnecessary procedures. On the ethical front, there is also a clear need for particularly detailed and comprehensive informed consent protocols, in which patients are adequately informed not only about the clinical significance of ctHPV DNA and its predictive capacity but also about the test’s limitations, possible false positives or negatives, and the psychological implications of persistent positivity [92–94]. Communication must therefore be clear, transparent, and tailored to the patient’s ability to understand their situation, considering their psychological and clinical status as well as their social context, supporting them through this process. It is essential to ensure that patients fully comprehend the risks and benefits associated with the use of this technology [87,95,96].

Medico-legal implications become particularly relevant in scenarios where ctHPV DNA results are discordant with conventional diagnostic tools. For example, a situation in which a patient presents with persistently positive ctHPV DNA but negative imaging may raise important questions about the most appropriate clinical management. Should invasive follow-up procedures be undertaken, or should surveillance be prioritized? If subsequent disease progression occurs, clinicians may face liability claims related either to overtreatment or to delayed diagnosis. In such cases, the issues outlined above become of vital importance. Patient information and informed decision-making are fundamental in the absence of definitive guidelines. When the clinical scenario is uncertain, management should be based on best medical practices and supported by a multidisciplinary approach. Patient care should thus result from teamwork involving different medical expertise, comprehensive information sharing, and active patient participation.

These scenarios also highlight how, even in gynecological settings, the presence of a medico-legal specialist within the multidisciplinary team may make a significant difference [97–99]. In conclusion, there is a clear need for standardized and internationally recognized guidelines for the interpretation and clinical integration of ctHPV DNA results.

Moreover, since ctHPV DNA represents highly sensitive genetic data, it is critical to guarantee rigorous privacy protection and compliance with current personal data protection regulations, such as the General Data Protection Regulation (GDPR) in the European Union [100]. The management and storage of biological samples and genetic information must be regulated to prevent unauthorized access and potential discrimination. Sharing such data for statistical and research purposes must also be consented to by the patient.

Furthermore, integrating ctHPV DNA into clinical practice and hospital protocols could provide valuable data to be used in updating oncological guidelines, accompanied by a revision of future related medico-legal responsibilities [101]. In this regard, it is necessary to clearly and jointly define the role of ctHPV DNA in diagnostic and therapeutic pathways, the criteria for appropriate use of the test, and the legal implications in case of omission or incorrect application, in order to protect both patients and healthcare professionals. Therefore, in the European Union, the GDPR establishes stringent requirements for the handling of genomic and biomarker data; By contrast, the regulatory framework in the United States, governed by the Health Insurance Portability and Accountability Act (HIPAA), places greater emphasis on data de-identification and the secure storage of health information, thereby allowing comparatively greater flexibility for research use [100].

In low-and middle-income countries (LMICs), where regulatory infrastructures may be less mature or inconsistently enforced, the challenges are twofold: ensuring adequate protection of patient confidentiality while simultaneously promoting the establishment of biobanks, research networks, and collaborative studies that are essential for scientific progress. This uneven regulatory landscape risks creating disparities in both patient protection and research opportunities.

Taken together, these differences highlight the pressing need to develop internationally shared ethical and regulatory frameworks. Such instruments should not only ensure the protection and confidentiality of data and safeguard patients’ rights [102–104], but also promote more equitable access to scientific collaborations and clinical innovations. In this way, technological progress can be reconciled with patient safety and privacy protection, ensuring uniform standards across heterogeneous healthcare systems.

An additional and crucial ethical consideration is represented by the cost-effectiveness and accessibility of ctHPV DNA testing, particularly in LMICs, where the burden of cervical cancer is highest. While advanced technologies such as digital droplet PCR and next-generation sequencing demonstrate high sensitivity, their widespread implementation is often constrained by costs, limited laboratory infrastructure, and shortages of trained personnel. Simplified, low-cost platforms or point-of-care adaptations may therefore be essential to ensure equitable access and maximize the global impact of ctHPV DNA testing.

From an ethical standpoint, the uneven availability of these technologies across income settings risks exacerbating existing global health disparities [105]. Patients in high-income countries are more likely to benefit from earlier detection and tailored disease management, whereas those in LMICs may remain reliant on less sensitive or delayed diagnostic approaches [106]. This disparity raises issues of distributive justice and highlights the moral obligation to reduce inequities in access to life-saving innovations. Addressing these gaps requires international collaboration, capacity-building efforts, and policy initiatives aimed at affordability and technology transfer, so that advances in ctHPV DNA testing contribute to narrowing rather than widening the divide between health systems [107,108].

### 4.4 Limitations

The current body of evidence on ctHPV DNA in cervical cancer is limited by significant methodological heterogeneity across studies. Differences in assay type (ddPCR vs. NGS-based platforms), analytic sensitivity, and genomic targets (E6/E7 vs. L1/L2 vs. integration sites) complicate direct comparison of detection rates and prognostic performance. Variability in sample type (plasma vs. serum) and pre-analytical processing introduces further inconsistencies in cfDNA yield and quality. In addition, patient populations differ substantially with respect to disease stage, HPV genotype distribution, and prior treatments, all of which influence ctHPV DNA detectability and kinetics. Sampling schedules and definitions of “persistent positivity” are not standardized, leading to variable estimates of lead time and prognostic value. Finally, most studies are single-center, include relatively small cohorts, and use different clinical endpoints, limiting generalizability and hindering pooled analyses. These limitations underscore the need for harmonized protocols, inter-laboratory validation, and large prospective multicenter trials to establish robust and clinically actionable thresholds for ctHPV DNA interpretation. Moreover, the findings of this review may have been influenced by selection bias. In particular, the exclusion of non-English language studies could have limited the scope of available evidence, potentially overlooking relevant data from regions where important research is published in other languages. Similarly, the decision to exclude conference abstracts may have led to the omission of more recent or preliminary findings, which might reflect ongoing developments in the field. These choices, while intended to ensure methodological rigor and data quality, may nonetheless have introduced a degree of bias and should be considered when interpreting the conclusions.

## 5 Conclusion and Future Questions

The study of circulating tumor HPV DNA in cervical cancer has seen promising advancements in recent decades. The distinct relationship between HPV and cervical cancer offers a unique opportunity to customize cancer care using precision medicine. Circulating tumor HPV DNA offers a non-invasive, sensitive, and specific means for assessing prognosis and monitoring for disease recurrence in this debilitating disease.

While we have described multiple promising studies in this paper demonstrating its utility, additional investigation needs to be conducted before this tool can be incorporated into routine clinical practice. Currently, there isn’t a validated biomarker for monitoring disease status in cervical cancer. We continue to use imaging to assess disease status. This is clearly limited by cost, practicality, and access to care. Furthermore, imaging requires that tumors reach a certain size prior to becoming visible. As cell-free DNA technology continues to become more sensitive, and as our ability to detect circulating tumor DNA at ever lower concentrations continues to improve, this technology has the potential to detect recurrences prior to a stage where even a recurrent cervical cancer can become curable. At this point, when a patient recurs centrally, with an isolated lesion, and has no signs of distant metastasis, pelvic exenteration can potentially become curative surgery. Indeed, the role of circulating tumor DNA in treatment selection, surgical or otherwise, remains to be elucidated. Do locally advanced cervical cancer patients, who have persistence of circulating tumor DNA after completion of chemotherapy and radiation, benefit from additional chemotherapy? Is there a level of circulating tumor DNA that can predict early cervical cancer patients who would benefit from foregoing surgery and are better suited for chemotherapy and radiation? Further research and clinical trials are crucial to establish standardized protocols and validate the utility of this technique in clinical practice. The potential for non-invasive monitoring of disease status offers improved quality of life to cancer survivors. As these ultrasensitive methods become more widely established, optimizing the cost of such technology will allow for dissemination to LMICs where the disease burden of cervical cancer is highest.

Optimizing sampling techniques using highly sensitive ddPCR and next-generation sequencing-based methods, larger prospective studies, and the use of standard processing techniques will further validate this marker for clinical use. At last, from a medico-legal perspective, although ctHPV DNA represents a promising frontier in the management of cervical cancer, its use must be accompanied by a careful evaluation of both medico-legal and ethical implications, so that such technological innovation translates into real clinical and societal benefits, while respecting patients’ rights and safety.

## Data Availability

Data available on request from the authors.
